# 4-[Bis(3,4-dimethoxy­phen­yl)meth­yl]pyridine ethanol monosolvate

**DOI:** 10.1107/S1600536810017460

**Published:** 2010-05-19

**Authors:** Fang-Fang Jian, Zhi-Peng Ni

**Affiliations:** a New Materials and Function Coordination Chemistry Laboratory, Qingdao University of Science and Technology, Qingdao 266042, People’s Republic of China

## Abstract

In the title compound, C_22_H_23_NO_4_·C_2_H_6_O, the pyridyl ring is aligned at 89.39 (2) and 87.41 (2)° with respect to the benzene rings, and the three rings connected to the methine C atom are arranged in a propeller-like conformation. The heterocycle is linked to the solvent mol­ecule by an O—H⋯N hydrogen bond.

## Related literature

For background to the use of pyridine and its derivatives as ligands to bridge different metal ions and form functional coordination compounds, see: Chen *et al.* (2007 ▶); Fasina *et al.* (2004 ▶); Mancisidor *et al.* (2008 ▶). For the synthesis, see: Ostaszewski (1998 ▶).
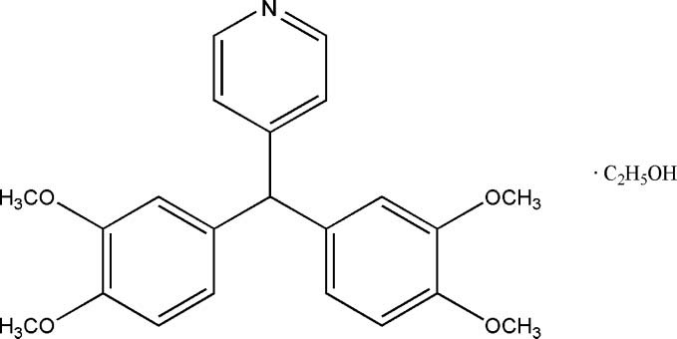


## Experimental

### 

#### Crystal data

C_22_H_23_NO_4_·C_2_H_6_O
*M* *_r_* = 411.48
Monoclinic, 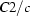

*a* = 29.564 (6) Å
*b* = 8.3810 (17) Å
*c* = 19.440 (4) Å
β = 107.94 (3)°
*V* = 4582.6 (18) Å^3^
*Z* = 8
Mo *K*α radiation
μ = 0.08 mm^−1^
*T* = 295 K
0.27 × 0.20 × 0.19 mm

#### Data collection

Enraf–Nonius CAD-4 diffractometer
14513 measured reflections
5573 independent reflections
2669 reflections with *I* > 2σ(*I*)
*R*_int_ = 0.042
3 standard reflections every 100 reflections  intensity decay: none

#### Refinement

*R*[*F* ^2^ > 2σ(*F* ^2^)] = 0.063
*wR*(*F* ^2^) = 0.174
*S* = 1.02
5573 reflections
277 parameters
H-atom parameters constrained
Δρ_max_ = 0.22 e Å^−3^
Δρ_min_ = −0.22 e Å^−3^

### 

Data collection: *CAD-4 Software* (Enraf–Nonius, 1989 ▶); cell refinement: *CAD-4 Software*; data reduction: *NRCVAX* (Gabe *et al.*, 1989 ▶); program(s) used to solve structure: *SHELXS97* (Sheldrick, 2008 ▶); program(s) used to refine structure: *SHELXL97* (Sheldrick, 2008 ▶); molecular graphics: *SHELXTL* (Sheldrick, 2008 ▶); software used to prepare material for publication: *WinGX* (Farrugia, 1999 ▶).

## Supplementary Material

Crystal structure: contains datablocks global, I. DOI: 10.1107/S1600536810017460/ng2771sup1.cif

Structure factors: contains datablocks I. DOI: 10.1107/S1600536810017460/ng2771Isup2.hkl

Additional supplementary materials:  crystallographic information; 3D view; checkCIF report

## Figures and Tables

**Table 1:** Hydrogen-bond geometry (Å, °)

*D*—H⋯*A*	*D*—H	H⋯*A*	*D*⋯*A*	*D*—H⋯*A*
O5—H5⋯N1	0.82	2.04	2.842 (4)	167

**Fig. 1.**
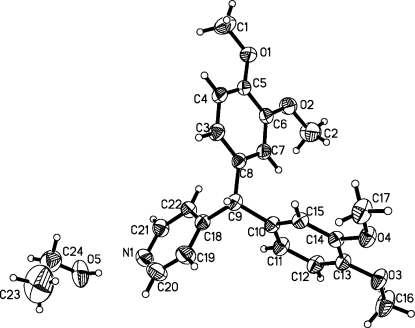
The molecular structure of the title compound with the atom-labeling scheme. Displacement ellipsoids are drawn at the 30% probability level.

## supplementary crystallographic information

### Comment

Pyridine and its derivatives, are of interest as ligands to bridge different metal ions to form functional coordination compounds, for example: 9,10-bis(4'- pyridylethynyl)-anthracene (Fasina *et al.*, 2004);2,6-bis-(imi-dazol-1 -yl)pyridine (Chen *et al.*, 2007); bis-(pyridine-2-ylmethyl)-benzylamine (Mancisidor *et al.*, 2008). In order to search for new pyridine compounds with higher bioactivity and optical properties, we synthesized the title compound.

In the title compound, the bond lengths and angles are generally normal. The dihedral angles between pyridine ring N1, C20, C19, C18, C22, C21(p1) with C3—C8 (p2) phenyl ring and C10—C15 (p3) phenyl ring are 89.39 (2)° and 87.41 (2)°, the dihedral angles between C3—C8 (p2) phenyl ring and C10—C15 (p3) phenyl ring is 84.33 (2)°, respectively.

The crystal structure is stabilized by intramolecular O—H···N hydrogen bonds (Table 1) and intramolecular C—H···O hydrogen bonds. The donor and acceptor distance are 3.4019Å for C(20) – H(20 A).. O(5) and 3.3902Å for C(21) – H(21 A).. O(3). In addition, there exist four kinds of C—H···Π interaction in the lattice [C2···Cg1=3.441 (2) Å; C3···Cg2=4.052 (3) Å; C17···Cg2=3.774 (2) Å; C24···Cg3=4.187 (1) Å; Cg1, Cg2 and Cg3 refer to pyridine, phenyl C3—C8 and phenyl ring C10—C15, respectively]. In the solid state, all above intermolecular interactions in the title compound stabilize the crystal packing structure.

### Experimental

The title compound was prepared by the reaction of 1,2-dimethoxybenzene (20 mmol), isonicotinaldehyde (40 mmol), and was stirred in dichloromethane solution with 84% sulfuric acid (10 ml) as activator (Ostaszewski *et al.*, 1998). Single crystals of the title compound suitable for X-ray measurements were obtained by recrystallization from ethanol at room temperature over a period of 3 days.

### Refinement

H atoms were positioned geometrically and treated as riding on their parent C atoms, with C—H distances in the range 0.93-0.97 Å, and with *U*_iso_(H)= 1.2-1.5*U*_eq_ of the parent atoms.

### Crystal data

**Table:**

C_22_H_23_NO_4_·C_2_H_6_O	*F*(000) = 1760
*M**_r_* = 411.48	*D*_x_ = 1.193 Mg m^−^^3^
Monoclinic, *C*2/*c*	Melting point: 342 K
Hall symbol: -C 2yc	Mo *K*α radiation, λ = 0.71073 Å
*a* = 29.564 (6) Å	Cell parameters from 25 reflections
*b* = 8.3810 (17) Å	θ = 1.5–25.5°
*c* = 19.440 (4) Å	µ = 0.08 mm^−^^1^
β = 107.94 (3)°	*T* = 295 K
*V* = 4582.6 (18) Å^3^	Block, colourless
*Z* = 8	0.27 × 0.20 × 0.19 mm

### Data collection

**Table:**

Enraf–Nonius CAD-4 diffractometer	*R*_int_ = 0.042
Radiation source: fine-focus sealed tube	θ_max_ = 28.3°, θ_min_ = 1.5°
graphite	*h* = −34→38
ω scans	*k* = −11→10
14513 measured reflections	*l* = −25→20
5573 independent reflections	3 standard reflections every 100 reflections
2669 reflections with *I* > 2σ(*I*)	intensity decay: none

### Refinement

**Table:**

Refinement on *F*^2^	Primary atom site location: structure-invariant direct methods
Least-squares matrix: full	Secondary atom site location: difference Fourier map
*R*[*F*^2^ > 2σ(*F*^2^)] = 0.063	Hydrogen site location: inferred from neighbouring sites
*wR*(*F*^2^) = 0.174	H-atom parameters constrained
*S* = 1.02	*w* = 1/[σ^2^(*F*_o_^2^) + (0.070*P*)^2^ + 0.6422*P*] where *P* = (*F*_o_^2^ + 2*F*_c_^2^)/3
5573 reflections	(Δ/σ)_max_ < 0.001
277 parameters	Δρ_max_ = 0.22 e Å^−^^3^
0 restraints	Δρ_min_ = −0.22 e Å^−^^3^

### Special details

**Table:**

Geometry. All esds (except the esd in the dihedral angle between two l.s. planes) are estimated using the full covariance matrix. The cell esds are taken into account individually in the estimation of esds in distances, angles and torsion angles; correlations between esds in cell parameters are only used when they are defined by crystal symmetry. An approximate (isotropic) treatment of cell esds is used for estimating esds involving l.s. planes.
Refinement. Refinement of *F*^2^ against ALL reflections. The weighted *R*-factor wR and goodness of fit *S* are based on *F*^2^, conventional *R*-factors *R* are based on *F*, with *F* set to zero for negative *F*^2^. The threshold expression of *F*^2^ > σ(*F*^2^) is used only for calculating *R*-factors(gt) etc. and is not relevant to the choice of reflections for refinement. *R*-factors based on *F*^2^ are statistically about twice as large as those based on *F*, and *R*- factors based on ALL data will be even larger.

### Fractional atomic coordinates and isotropic or equivalent isotropic displacement parameters (Å^2^)

**Table:**

	*x*	*y*	*z*	*U*_iso_*/*U*_eq_	
O1	0.30498 (6)	1.1065 (2)	0.93614 (10)	0.0848 (6)	
O2	0.23104 (6)	1.2671 (2)	0.86968 (10)	0.0795 (5)	
O3	0.07275 (6)	1.2438 (2)	0.48851 (8)	0.0711 (5)	
O4	0.15262 (5)	1.1115 (2)	0.49173 (8)	0.0648 (5)	
O5	0.02886 (9)	0.3191 (3)	0.87938 (15)	0.1157 (8)	
H5	0.0360	0.3742	0.8494	0.139*	
N1	0.06698 (8)	0.5258 (3)	0.79531 (13)	0.0770 (6)	
C1	0.34474 (13)	1.0218 (4)	0.97716 (19)	0.1275 (14)	
H1B	0.3659	1.0926	1.0109	0.191*	
H1C	0.3608	0.9765	0.9458	0.191*	
H1D	0.3349	0.9380	1.0030	0.191*	
C2	0.19141 (11)	1.3623 (4)	0.83401 (19)	0.1037 (11)	
H2A	0.1957	1.4676	0.8544	0.156*	
H2B	0.1631	1.3158	0.8399	0.156*	
H2C	0.1884	1.3683	0.7835	0.156*	
C3	0.24084 (8)	0.8041 (3)	0.80555 (12)	0.0592 (6)	
H3A	0.2443	0.6994	0.7922	0.071*	
C4	0.27668 (8)	0.8747 (3)	0.86124 (13)	0.0627 (7)	
H4A	0.3039	0.8167	0.8845	0.075*	
C5	0.27240 (8)	1.0275 (3)	0.88221 (12)	0.0573 (6)	
C6	0.23211 (8)	1.1147 (3)	0.84613 (12)	0.0550 (6)	
C7	0.19656 (8)	1.0447 (3)	0.79090 (12)	0.0546 (6)	
H7A	0.1696	1.1034	0.7672	0.066*	
C8	0.20042 (7)	0.8876 (3)	0.77007 (11)	0.0491 (5)	
C9	0.16086 (7)	0.8074 (3)	0.71137 (10)	0.0503 (6)	
H9A	0.1764	0.7297	0.6884	0.060*	
C10	0.13455 (7)	0.9216 (3)	0.65203 (11)	0.0493 (5)	
C11	0.09260 (8)	0.9934 (3)	0.64980 (12)	0.0608 (7)	
H11A	0.0783	0.9687	0.6849	0.073*	
C12	0.07107 (8)	1.1021 (3)	0.59624 (12)	0.0626 (7)	
H12A	0.0427	1.1508	0.5960	0.075*	
C13	0.09124 (8)	1.1383 (3)	0.54365 (11)	0.0528 (6)	
C14	0.13426 (7)	1.0673 (3)	0.54553 (11)	0.0481 (5)	
C15	0.15522 (7)	0.9611 (3)	0.59909 (11)	0.0486 (5)	
H15A	0.1839	0.9142	0.6002	0.058*	
C16	0.02736 (11)	1.3087 (4)	0.48086 (18)	0.1096 (12)	
H16A	0.0186	1.3801	0.4403	0.164*	
H16B	0.0282	1.3659	0.5240	0.164*	
H16C	0.0045	1.2240	0.4731	0.164*	
C17	0.19682 (9)	1.0437 (3)	0.49298 (14)	0.0800 (8)	
H17A	0.2065	1.0867	0.4540	0.120*	
H17B	0.1935	0.9300	0.4877	0.120*	
H17C	0.2203	1.0683	0.5382	0.120*	
C18	0.12750 (8)	0.7117 (3)	0.74155 (11)	0.0517 (6)	
C19	0.09979 (10)	0.5923 (3)	0.70163 (13)	0.0783 (8)	
H19A	0.1008	0.5715	0.6551	0.094*	
C20	0.07074 (11)	0.5034 (4)	0.72947 (17)	0.0918 (9)	
H20A	0.0527	0.4231	0.7009	0.110*	
C21	0.09305 (9)	0.6417 (3)	0.83336 (14)	0.0657 (7)	
H21A	0.0909	0.6613	0.8793	0.079*	
C22	0.12338 (8)	0.7359 (3)	0.80920 (12)	0.0566 (6)	
H22A	0.1410	0.8157	0.8388	0.068*	
C23	0.0512 (2)	0.0797 (6)	0.8412 (3)	0.227 (3)	
H23A	0.0652	−0.0205	0.8605	0.340*	
H23B	0.0658	0.1179	0.8066	0.340*	
H23C	0.0177	0.0656	0.8181	0.340*	
C24	0.05815 (13)	0.1877 (5)	0.8959 (2)	0.1247 (13)	
H24A	0.0909	0.2236	0.9096	0.150*	
H24B	0.0534	0.1345	0.9374	0.150*	

### Atomic displacement parameters (Å^2^)

**Table:**

	*U*^11^	*U*^22^	*U*^33^	*U*^12^	*U*^13^	*U*^23^
O1	0.0727 (12)	0.0672 (12)	0.0895 (12)	−0.0157 (10)	−0.0119 (10)	0.0122 (10)
O2	0.0763 (13)	0.0534 (11)	0.0964 (13)	−0.0007 (9)	0.0085 (10)	−0.0037 (10)
O3	0.0654 (11)	0.0860 (12)	0.0681 (10)	0.0276 (10)	0.0297 (8)	0.0295 (9)
O4	0.0664 (11)	0.0799 (12)	0.0586 (9)	0.0179 (9)	0.0346 (8)	0.0171 (8)
O5	0.1304 (19)	0.0829 (16)	0.164 (2)	0.0193 (15)	0.0893 (17)	0.0368 (15)
N1	0.0741 (15)	0.0770 (16)	0.0810 (15)	−0.0157 (13)	0.0257 (12)	0.0058 (13)
C1	0.105 (3)	0.105 (3)	0.121 (3)	−0.007 (2)	−0.041 (2)	0.014 (2)
C2	0.089 (2)	0.0649 (19)	0.142 (3)	0.0179 (17)	0.013 (2)	−0.011 (2)
C3	0.0556 (15)	0.0595 (15)	0.0647 (14)	0.0085 (12)	0.0216 (12)	0.0095 (12)
C4	0.0476 (14)	0.0656 (17)	0.0708 (16)	0.0053 (12)	0.0124 (12)	0.0195 (14)
C5	0.0500 (14)	0.0582 (16)	0.0595 (14)	−0.0057 (12)	0.0107 (11)	0.0165 (12)
C6	0.0540 (15)	0.0517 (15)	0.0606 (14)	−0.0043 (12)	0.0193 (12)	0.0083 (12)
C7	0.0482 (14)	0.0567 (15)	0.0602 (14)	0.0032 (11)	0.0185 (11)	0.0127 (12)
C8	0.0457 (13)	0.0568 (15)	0.0484 (12)	0.0034 (11)	0.0198 (10)	0.0106 (11)
C9	0.0535 (13)	0.0549 (14)	0.0457 (12)	0.0080 (11)	0.0201 (10)	0.0036 (10)
C10	0.0512 (13)	0.0551 (14)	0.0438 (11)	0.0057 (11)	0.0179 (10)	0.0021 (10)
C11	0.0583 (15)	0.0809 (18)	0.0514 (13)	0.0138 (13)	0.0289 (11)	0.0143 (12)
C12	0.0506 (14)	0.0802 (18)	0.0621 (14)	0.0187 (13)	0.0247 (12)	0.0134 (13)
C13	0.0518 (14)	0.0593 (14)	0.0481 (12)	0.0068 (11)	0.0163 (10)	0.0071 (11)
C14	0.0488 (13)	0.0541 (13)	0.0448 (11)	0.0023 (11)	0.0194 (10)	−0.0013 (10)
C15	0.0458 (12)	0.0539 (14)	0.0486 (12)	0.0060 (11)	0.0185 (10)	−0.0020 (11)
C16	0.086 (2)	0.139 (3)	0.117 (2)	0.061 (2)	0.0505 (19)	0.065 (2)
C17	0.0815 (19)	0.100 (2)	0.0775 (17)	0.0284 (17)	0.0530 (15)	0.0195 (16)
C18	0.0519 (13)	0.0559 (14)	0.0465 (12)	0.0020 (11)	0.0141 (10)	0.0031 (11)
C19	0.094 (2)	0.088 (2)	0.0525 (14)	−0.0251 (17)	0.0221 (14)	−0.0119 (14)
C20	0.097 (2)	0.094 (2)	0.081 (2)	−0.0386 (18)	0.0223 (17)	−0.0097 (17)
C21	0.0691 (17)	0.0713 (17)	0.0635 (15)	−0.0018 (15)	0.0304 (13)	0.0059 (14)
C22	0.0624 (15)	0.0562 (14)	0.0543 (13)	−0.0049 (12)	0.0224 (11)	−0.0031 (11)
C23	0.330 (9)	0.152 (5)	0.213 (6)	0.083 (6)	0.107 (6)	−0.038 (4)
C24	0.090 (3)	0.126 (4)	0.152 (4)	0.010 (2)	0.029 (2)	0.031 (3)

### Geometric parameters (Å, °)

**Table:**

O1—C5	1.358 (3)	C9—H9A	0.9800
O1—C1	1.395 (3)	C10—C11	1.367 (3)
O2—C6	1.361 (3)	C10—C15	1.389 (3)
O2—C2	1.410 (3)	C11—C12	1.383 (3)
O3—C13	1.367 (3)	C11—H11A	0.9300
O3—C16	1.413 (3)	C12—C13	1.367 (3)
O4—C14	1.369 (2)	C12—H12A	0.9300
O4—C17	1.418 (3)	C13—C14	1.395 (3)
O5—C24	1.377 (4)	C14—C15	1.365 (3)
O5—H5	0.8200	C15—H15A	0.9300
N1—C21	1.317 (3)	C16—H16A	0.9600
N1—C20	1.332 (3)	C16—H16B	0.9600
C1—H1B	0.9600	C16—H16C	0.9600
C1—H1C	0.9600	C17—H17A	0.9600
C1—H1D	0.9600	C17—H17B	0.9600
C2—H2A	0.9600	C17—H17C	0.9600
C2—H2B	0.9600	C18—C19	1.373 (3)
C2—H2C	0.9600	C18—C22	1.373 (3)
C3—C8	1.373 (3)	C19—C20	1.368 (4)
C3—C4	1.392 (3)	C19—H19A	0.9300
C3—H3A	0.9300	C20—H20A	0.9300
C4—C5	1.362 (3)	C21—C22	1.381 (3)
C4—H4A	0.9300	C21—H21A	0.9300
C5—C6	1.389 (3)	C22—H22A	0.9300
C6—C7	1.381 (3)	C23—C24	1.362 (5)
C7—C8	1.392 (3)	C23—H23A	0.9600
C7—H7A	0.9300	C23—H23B	0.9600
C8—C9	1.517 (3)	C23—H23C	0.9600
C9—C10	1.516 (3)	C24—H24A	0.9700
C9—C18	1.522 (3)	C24—H24B	0.9700
			
C5—O1—C1	117.8 (2)	C11—C12—H12A	119.9
C6—O2—C2	117.9 (2)	C12—C13—O3	124.7 (2)
C13—O3—C16	117.95 (19)	C12—C13—C14	119.4 (2)
C14—O4—C17	117.27 (18)	O3—C13—C14	115.96 (19)
C24—O5—H5	109.5	C15—C14—O4	124.44 (19)
C21—N1—C20	115.9 (2)	C15—C14—C13	119.63 (19)
O1—C1—H1B	109.5	O4—C14—C13	115.93 (19)
O1—C1—H1C	109.5	C14—C15—C10	121.4 (2)
H1B—C1—H1C	109.5	C14—C15—H15A	119.3
O1—C1—H1D	109.5	C10—C15—H15A	119.3
H1B—C1—H1D	109.5	O3—C16—H16A	109.5
H1C—C1—H1D	109.5	O3—C16—H16B	109.5
O2—C2—H2A	109.5	H16A—C16—H16B	109.5
O2—C2—H2B	109.5	O3—C16—H16C	109.5
H2A—C2—H2B	109.5	H16A—C16—H16C	109.5
O2—C2—H2C	109.5	H16B—C16—H16C	109.5
H2A—C2—H2C	109.5	O4—C17—H17A	109.5
H2B—C2—H2C	109.5	O4—C17—H17B	109.5
C8—C3—C4	120.7 (2)	H17A—C17—H17B	109.5
C8—C3—H3A	119.7	O4—C17—H17C	109.5
C4—C3—H3A	119.7	H17A—C17—H17C	109.5
C5—C4—C3	120.9 (2)	H17B—C17—H17C	109.5
C5—C4—H4A	119.5	C19—C18—C22	115.8 (2)
C3—C4—H4A	119.5	C19—C18—C9	120.7 (2)
O1—C5—C4	125.5 (2)	C22—C18—C9	123.4 (2)
O1—C5—C6	115.3 (2)	C20—C19—C18	120.8 (2)
C4—C5—C6	119.2 (2)	C20—C19—H19A	119.6
O2—C6—C7	124.7 (2)	C18—C19—H19A	119.6
O2—C6—C5	115.4 (2)	N1—C20—C19	123.5 (3)
C7—C6—C5	119.9 (2)	N1—C20—H20A	118.2
C6—C7—C8	121.1 (2)	C19—C20—H20A	118.2
C6—C7—H7A	119.4	N1—C21—C22	124.0 (2)
C8—C7—H7A	119.4	N1—C21—H21A	118.0
C3—C8—C7	118.2 (2)	C22—C21—H21A	118.0
C3—C8—C9	120.1 (2)	C18—C22—C21	120.0 (2)
C7—C8—C9	121.67 (19)	C18—C22—H22A	120.0
C10—C9—C8	112.84 (19)	C21—C22—H22A	120.0
C10—C9—C18	112.59 (18)	C24—C23—H23A	109.5
C8—C9—C18	112.63 (16)	C24—C23—H23B	109.5
C10—C9—H9A	106.0	H23A—C23—H23B	109.5
C8—C9—H9A	106.0	C24—C23—H23C	109.5
C18—C9—H9A	106.0	H23A—C23—H23C	109.5
C11—C10—C15	118.3 (2)	H23B—C23—H23C	109.5
C11—C10—C9	123.36 (19)	C23—C24—O5	114.7 (4)
C15—C10—C9	118.31 (19)	C23—C24—H24A	108.6
C10—C11—C12	121.1 (2)	O5—C24—H24A	108.6
C10—C11—H11A	119.4	C23—C24—H24B	108.6
C12—C11—H11A	119.4	O5—C24—H24B	108.6
C13—C12—C11	120.2 (2)	H24A—C24—H24B	107.6
C13—C12—H12A	119.9		
			
C8—C3—C4—C5	0.5 (3)	C10—C11—C12—C13	0.8 (4)
C1—O1—C5—C4	−4.8 (4)	C11—C12—C13—O3	180.0 (2)
C1—O1—C5—C6	176.1 (3)	C11—C12—C13—C14	−1.5 (4)
C3—C4—C5—O1	179.4 (2)	C16—O3—C13—C12	−6.5 (4)
C3—C4—C5—C6	−1.5 (3)	C16—O3—C13—C14	174.9 (2)
C2—O2—C6—C7	−1.8 (4)	C17—O4—C14—C15	−1.1 (3)
C2—O2—C6—C5	178.7 (2)	C17—O4—C14—C13	178.8 (2)
O1—C5—C6—O2	0.3 (3)	C12—C13—C14—C15	1.0 (3)
C4—C5—C6—O2	−178.9 (2)	O3—C13—C14—C15	179.7 (2)
O1—C5—C6—C7	−179.3 (2)	C12—C13—C14—O4	−178.9 (2)
C4—C5—C6—C7	1.5 (3)	O3—C13—C14—O4	−0.2 (3)
O2—C6—C7—C8	−180.0 (2)	O4—C14—C15—C10	−180.0 (2)
C5—C6—C7—C8	−0.5 (3)	C13—C14—C15—C10	0.1 (3)
C4—C3—C8—C7	0.6 (3)	C11—C10—C15—C14	−0.7 (3)
C4—C3—C8—C9	−177.74 (19)	C9—C10—C15—C14	−177.8 (2)
C6—C7—C8—C3	−0.6 (3)	C10—C9—C18—C19	72.2 (3)
C6—C7—C8—C9	177.71 (19)	C8—C9—C18—C19	−158.8 (2)
C3—C8—C9—C10	−147.48 (19)	C10—C9—C18—C22	−108.8 (2)
C7—C8—C9—C10	34.3 (3)	C8—C9—C18—C22	20.2 (3)
C3—C8—C9—C18	83.7 (2)	C22—C18—C19—C20	−0.9 (4)
C7—C8—C9—C18	−94.6 (2)	C9—C18—C19—C20	178.2 (3)
C8—C9—C10—C11	−97.2 (3)	C21—N1—C20—C19	0.5 (4)
C18—C9—C10—C11	31.7 (3)	C18—C19—C20—N1	0.4 (5)
C8—C9—C10—C15	79.6 (2)	C20—N1—C21—C22	−0.9 (4)
C18—C9—C10—C15	−151.5 (2)	C19—C18—C22—C21	0.5 (3)
C15—C10—C11—C12	0.2 (4)	C9—C18—C22—C21	−178.5 (2)
C9—C10—C11—C12	177.1 (2)	N1—C21—C22—C18	0.4 (4)

### Hydrogen-bond geometry (Å, °)

**Table:**

*D*—H···*A*	*D*—H	H···*A*	*D*···*A*	*D*—H···*A*
O5—H5···N1	0.82	2.04	2.842 (4)	167
